# How whales used to filter: exceptionally preserved baleen in a Miocene cetotheriid

**DOI:** 10.1111/joa.12622

**Published:** 2017-05-24

**Authors:** Felix G. Marx, Alberto Collareta, Anna Gioncada, Klaas Post, Olivier Lambert, Elena Bonaccorsi, Mario Urbina, Giovanni Bianucci

**Affiliations:** ^1^ School of Biological Sciences Monash University Clayton Vic. Australia; ^2^ Geosciences Museum Victoria Melbourne Vic. Australia; ^3^ D.O. Terre et Histoire de la Vie Institut Royal des Sciences Naturelles de Belgique Brussels Belgium; ^4^ Dipartimento di Scienze della Terra Università di Pisa Pisa Italy; ^5^ Dottorato Regionale in Scienze della Terra Pegaso Pisa Italy; ^6^ Natuurhistorisch Museum Rotterdam Rotterdam The Netherlands; ^7^ Departamento de Paleontología de Vertebrados Museo de Historia Natural de la Universidad Nacional Mayor de San Marcos Lima Peru

**Keywords:** baleen whale, Cetotheriidae, filter feeding, Mysticeti, *Piscobalaena*, suction feeding

## Abstract

Baleen is a comb‐like structure that enables mysticete whales to bulk feed on vast quantities of small prey, and ultimately allowed them to become the largest animals on Earth. Because baleen rarely fossilises, extremely little is known about its evolution, structure and function outside the living families. Here we describe, for the first time, the exceptionally preserved baleen apparatus of an entirely extinct mysticete morphotype: the Late Miocene cetotheriid, *Piscobalaena nana*, from the Pisco Formation of Peru. The baleen plates of *P. nana* are closely spaced and built around relatively dense, fine tubules, as in the enigmatic pygmy right whale, *Caperea marginata*. Phosphatisation of the intertubular horn, but not the tubules themselves, suggests *in vivo* intertubular calcification. The size of the rack matches the distribution of nutrient foramina on the palate, and implies the presence of an unusually large subrostral gap. Overall, the baleen morphology of *Piscobalaena* likely reflects the interacting effects of size, function and phylogeny, and reveals a previously unknown degree of complexity in modern mysticete feeding evolution.

## Introduction

Baleen is the key adaptation that allows mysticetes to filter small prey directly from seawater, and thus is central to understanding their ecology and evolution (Pivorunas, 1979). Because of its keratinous nature, baleen generally decays along with the remainder of the soft tissue. Descriptions of fossilised baleen are rare, and currently restricted to specimens (e.g. fossil rorquals) that closely resemble extant whales in their morphology and, presumably, lifestyle (Esperante et al. 2008; Bisconti, 2012; Gioncada et al. 2016; Marx & Kohno, 2016). By contrast, little is known about baleen structure and function in extinct morphotypes that substantially differ from the living species in their overall anatomy, which presents a major obstacle to understanding the evolution of mysticete feeding ecology.

**Figure 1:**
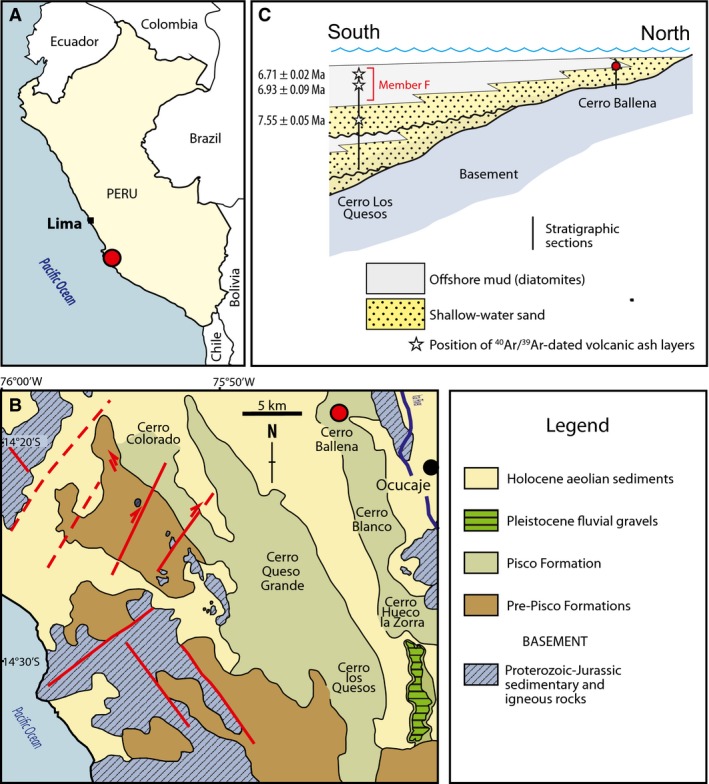
Location and geological overview of Cerro Ballena. (A) Location of Cerro Ballena within Peru; (B) geological map showing the regional extent of the Pisco Formation and the location of Cerro Ballena relative to the better‐known localities of Cerro Los Quesos and Cerro Colorado; (C) stratigraphic overview of the Pisco Formation at Cerro Ballena and the nearby locality of Cerro Los Quesos, including relevant ^40^Ar/^39^Ar dates (Di Celma et al. 2016).

Here, we describe the exceptionally preserved baleen of a new specimen of *Piscobalaena nana*, a small Late Miocene mysticete from the coastal deserts of Peru (Fig. 1). *Piscobalaena* belongs to the Cetotheriidae (Bouetel & de Muizon, 2006), an iconic family that until recently had been considered extinct, but has now – controversially (Bisconti, 2015) – been proposed to include the living pygmy right whale, *Caperea marginata* (Marx & Fordyce, 2016). Crucially, *Piscobalaena* is also a ‘typical’ cetotheriid (unlike the highly autapomorphic *Caperea* and its fossil relative, *Miocaperea*), and therefore representative of a major morphotype no longer present in the modern oceans. *Piscobalaena* thus offers the first opportunity to chart the evolution of the defining feature of baleen whales beyond the confines of the extant lineages.

## Materials and methods

### Materials and geological setting

**Figure 2:**
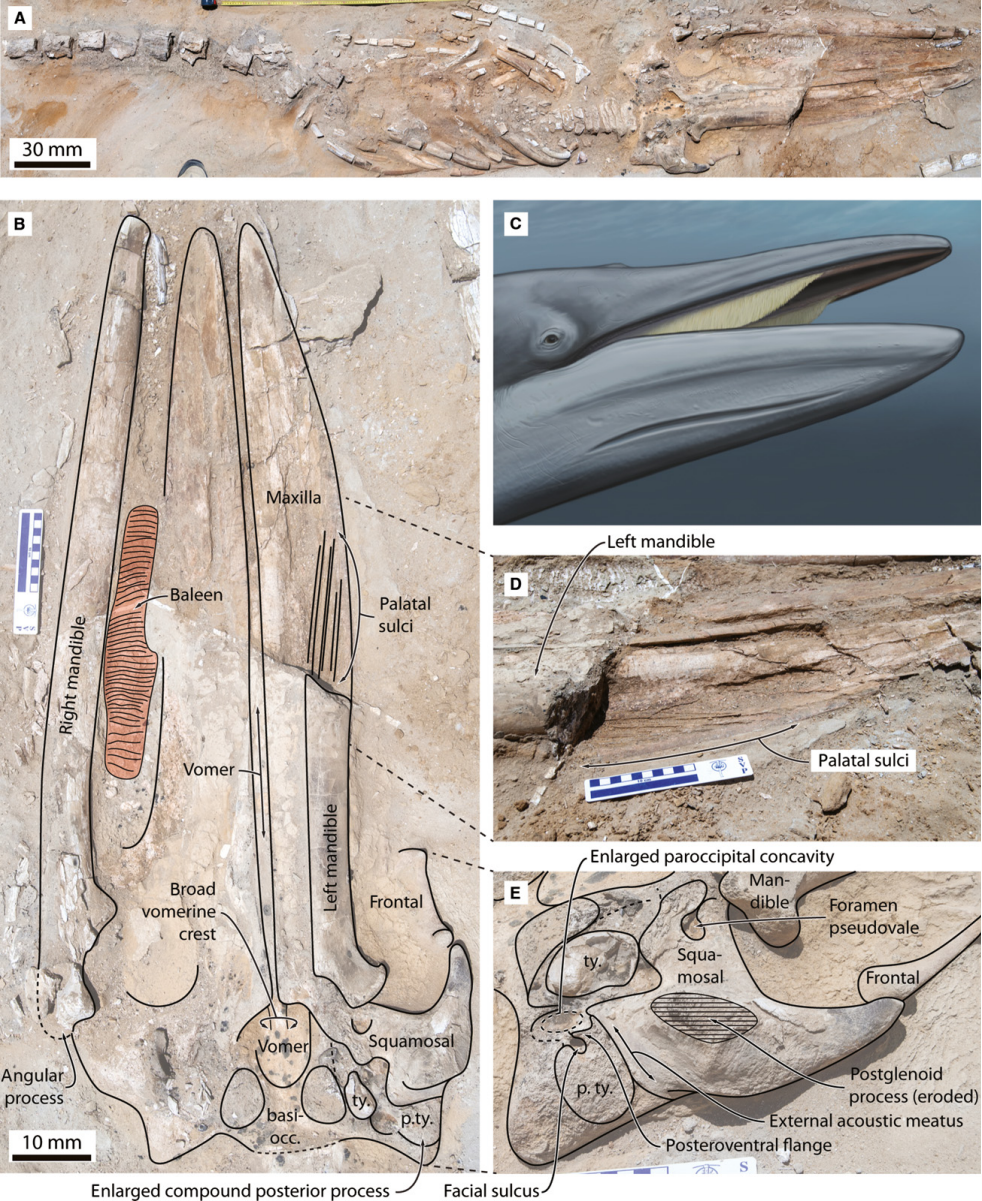
*Piscobalaena nana* (MUSM 3292) preserving baleen. (A) Overview of *in situ* skeleton, in ventral view; (B) explanatory drawing of skull in ventral view, highlighting relevant morphological features and the position of the baleen rack; (C) life reconstruction of *Piscobalaena*, showing the large subrostral gap; (D) close‐up of the left maxilla in ventrolateral view, showing details of the palatal sulci; (E) close‐up of the auditory region in posterolateral view, showing the enlarged compound posterior process and ventrally floored facial sulcus. Life reconstruction by Carl Buell. See Figs S1 and S2 for larger, unlabelled versions of (B) and (E). Abbreviations: basiocc., basioccipital; con., mandibular condyle; p. ty., compound posterior process of the tympanoperiotic; ty., tympanic bulla.

The baleen described here formed part of an articulated skeleton, preserved ventral side up (Fig. 2A). Because of advanced erosion of the bones and limited resources, only the baleen rack itself was collected and accessioned at MUSM (specimen 3292). The specimen was recovered from an exposure of the Pisco Formation at Cerro Ballena, a rocky hill located about 3 km west of the village of Ocucaje, Ica District, Peru (S 14°20′51.5″, W 75°42′36.1″; Fig. 1). Note that this site is distinct from the locality of the same name in the Atacama Region of Chile (Pyenson et al. 2014). The Pisco Formation is a highly fossiliferous, Neogene deposit exposed along the southern coast of Peru. In the East Pisco Basin, the formation consists mainly of Miocene shallow marine deposits, including conglomerates, sandstones, diatomites, diatomaceous siltstones, tuffaceous beds and dolomitic horizons, which are thought to reflect strong coastal upwelling and high ocean primary productivity (Suess et al. 1988; Dunbar et al. 1990; Brand et al. 2004).

The skeleton was embedded in fine‐grained, poorly cemented diatomaceous siltstone with no obvious sedimentary structures, and was found above the M10 marker bed as defined by Brand et al. (2011). The latter corresponds to the P2‐8 horizon of Di Celma et al. (2017), and is located above a more widely distributed horizon termed P2‐6 or the ‘Flor’ marker bed (Bianucci et al. 2016; Di Celma et al. 2017). At the highly fossiliferous locality of Cerro Los Quesos, the Flor marker bed defines the lower boundary of ‘Member F’, which corresponds to the uppermost sediment package of the P2 sequence (Di Celma et al. 2017), and includes two volcanic ash layers that have been ^40^Ar/^39^Ar‐dated to 6.93 ± 0.09 Ma and ≥ 6.71 ± 0.02 Ma, respectively (Di Celma et al. 2016; Gariboldi et al. 2017; Fig. 1). Below, the age of ‘Member F’ is constrained by a further ^40^Ar/^39^Ar date of 7.55 ± 0.05 Ma. These estimates match unpublished ^40^Ar/^39^Ar dates of 6.94–6.43 Ma cited for Cerro Ballena by Esperante et al. (2015), and suggest an age of approximately 7.6–6.4 Ma for our specimen.

### Scanning electron microscopy‐energy‐dispersive X‐ray spectroscopy (SEM‐EDS) analyses

Small fragments of the fossil baleen were carbon‐coated for SEM‐EDS with a Philips XL30 SEM equipped with DX4i EDAX microanalysis, housed at the University of Pisa (Italy). Analytical conditions were: 20 kV accelerating voltage; 5 nA beam current. SEM‐EDS was carried out both via spot analysis of single, large crystals, and within 5 × 5 μm or 10 × 10 μm raster areas. Imaging was carried out with secondary electrons and backscattered electrons.

### X‐ray diffraction analysis

Some phosphatic material was manually collected with a needle and powdered for X‐ray powder diffraction analysis (XRPD), using a Bruker D2 Phaser diffractometer operating at 10 mA and 30 kV, a flat background‐free sample holder, and Cu Kα_1_ radiation with λ = 1.54060 Å. Data were processed using the software DIFFRAC.EVA V4.1, and the peaks indexed on the basis of fluorapatite with a hexagonal unit cell (*a *= 9.3465, *c *= 6.8928 Å).

### Institutional abbreviations

IRSNB, Institut royal des Sciences naturelles de Belgique, Brussels, Belgium; MNHN, Muséum National d'Histoire Naturelle, Paris, France; MUSM, Museo de Historia Natural de la Universidad Nacional Mayor de San Marcos, Lima, Peru; SMNK, Staatliches Museum für Naturkunde, Karlsruhe, Germany; USNM, National Museum of Natural History, Smithsonian Institution, Washington, DC, USA.

## Results

MUSM 3292 is a cetotheriid based on the presence of: (i) a distally expanded compound posterior process of the tympanoperiotic; (ii) an anteriorly expanded paroccipital concavity flooring the facial sulcus via a distinct posteroventral flange; (iii) a low and broadly triangular coronoid process of the mandible; and (iv) a posteriorly extended angular process of the mandible (Whitmore & Barnes, 2008; El Adli et al. 2014; Marx & Fordyce, 2016); and belongs to *P. nana* (the most common cetotheriid in this area and time period) based on the broad exposure of the vomer on the palate, and the wide and transversely rounded vomerine crest (Bouetel & de Muizon, 2006; Fig. 2). Its relatively large size (condylobasal length = 1180 mm; bizygomatic width = 460 mm) and firmly attached vertebral epiphyses identify the specimen as an adult (Moran et al. 2015).

The right baleen rack is preserved, but detached from the palate, displaced laterally, and partially draped over the right mandible (Fig. 2). Horizontally oriented striations on the individual plates, here interpreted as traces of tubules (see below), suggest that the rack has turned outwards by approximately 90 °. As preserved, the rack comprises 54 plates and is relatively short, terminating approximately halfway along the rostrum. *Post‐mortem* distortion has likely affected plate density. Nevertheless, regular plate spacing and the presence of sediment infill between individual laminae suggest that the rack has retained at least some of its original shape, permitting a tentative estimate.

**Figure 3:**
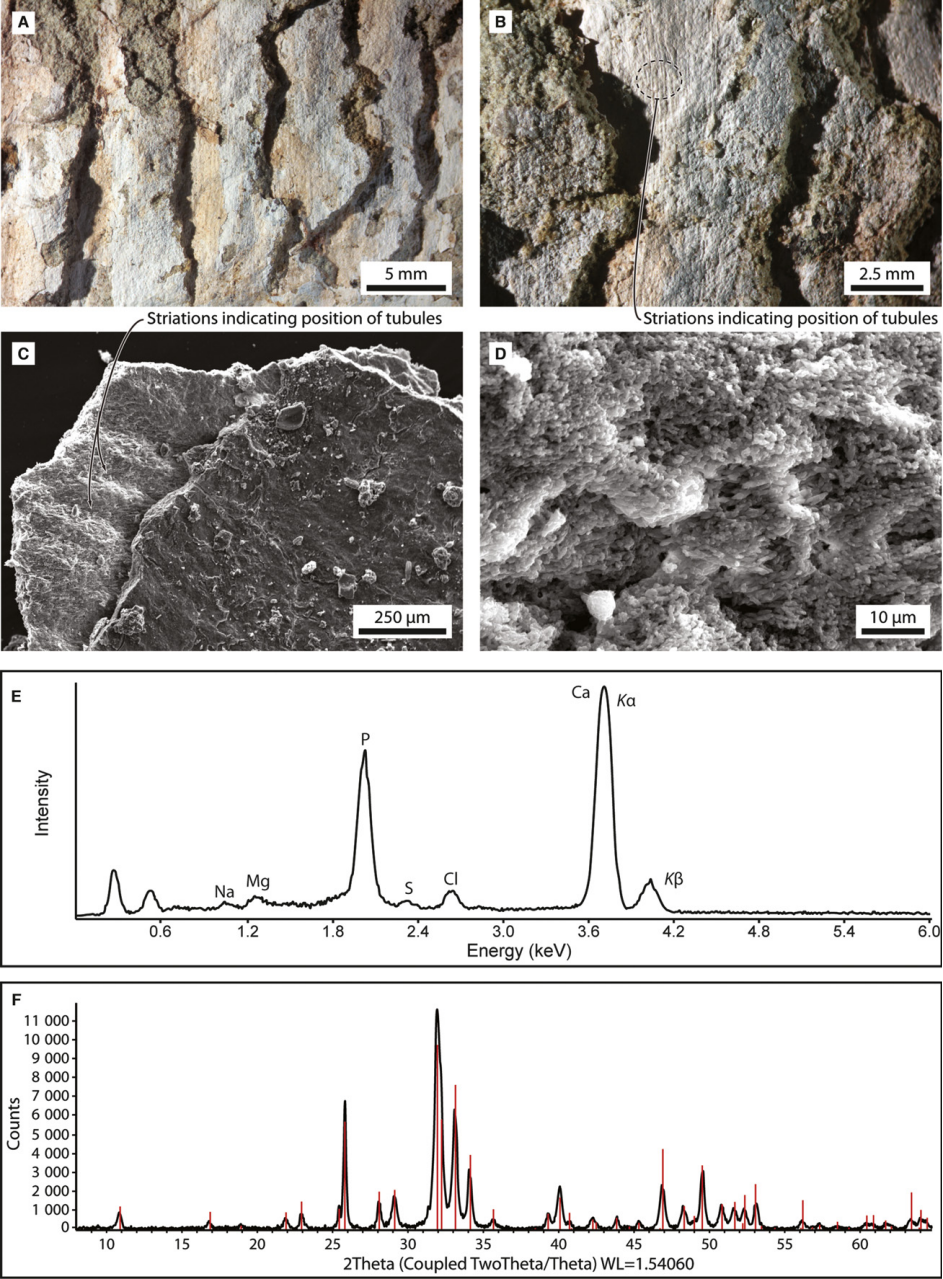
Phosphatised baleen of *Piscobalaena nana* (MUSM 3292). (A, B) Close‐ups of a short section of the right rack; (C) scanning electron microscope (SEM) image of a single plate, with striations indicating the location of decayed tubules, (D) close‐up of (C) showing fluorapatite crystals (ranging from < 1 to 5 μm) forming honeycomb‐like structures inside the plate; (E) Energy‐dispersive X‐ray spectroscopy (EDS) spectrum of one of the fossil baleen plates (5 × 5 μm raster area, interior of the plate), indicating Ca‐P chemistry; (F) X‐ray powder diffraction analysis (XRPD) pattern of the phosphatic material forming the fossil baleen plates; the red lines represent the position and intensity of the diffraction peaks of fluorapatite, which closely match the experimental diffraction pattern.

Plate density is about 4 plates cm^−1^ if measured based on intraplate distance (i.e. perpendicular to the plate surface), but 1.8 plates cm^−1^ if measured parasagittally, with neither estimate being entirely reliable owing to dorsoventral compression of the rack. A conservative compromise, allowing for one extra millimetre per plate to account for decay, places plate density about 2 plates cm^−1^ – higher than in most living baleen whales, but comparable to *Caperea*,* Miocaperea*, the extant rorquals *Balaenoptera acutorostrata* and *B. borealis* and, possibly, certain fossil balaenopterids (Bisconti, 2012; Young, 2012; Gioncada et al. 2016; Marx & Kohno, 2016). The plates themselves are well preserved (Fig. 3A–D) and consist entirely of Ca‐phosphate, identified as fluorapatite (Fig. 3E,F). Under magnification, the inside of each plate reveals a honeycomb‐like structure composed of relatively large crystals (Fig. 3D). The cavities within the honeycomb average 10 μm in size, with the overall pattern recalling the arrangement of cells in the intertubular horn (Fudge et al. 2009: fig. 33).

Parallel striations inside the plates likely represent the original locations of the tubules (Fig. 3B,C), implying a density of 80–100 tubules cm^−1^, and a tubule diameter of 0.10–0.13 mm. These estimates are more reliable than plate density, as the internal structure of the plates is more resistant to compression and does not appear to have been obviously distorted. Similar densities and diameters occur in *Caperea*, and the right whales *Eubalaena glacialis* and (tubule diameter only) *Balaena mysticetus* (Young, 2012). Unlike in a previously described balaenopterid from Peru (Gioncada et al. 2016), the tubules themselves appear entirely decayed.

## Discussion

### Exceptional preservation

MUSM 3293 shows an exceptional degree of preservation thanks to phosphatisation of the right baleen rack. Phosphatisation of soft tissues with fine replication of microstructures implies the development of localised conditions suitable for apatite nucleation (Schiffbauer et al. 2014), with a microbially induced anoxic milieu and a decay‐related decrease in pH favouring apatite over Ca‐Mg‐Fe carbonates (Briggs et al. 1993; Trinajstic et al. 2007). The preservation of the intertubular horn, but not the tubules, likely reflects *in vivo* calcification patterns, with hydroxyapatite occurring mainly outside the tubules. Similar calcification of the intertubular horn also occurs in *B. acutorostrata*, but detailed comparative data are currently lacking for most extant species (Szewciw et al. 2010). Following death, the intertubular crystallites may have helped to retard the decay of the plates (Gioncada et al. 2016), thereby facilitating Ca‐phosphate mineralization (Fig. 3E) and leading to cellular‐level preservation (Xiao & Schiffbauer, 2008).

### Size of the baleen rack

Anatomically, one of the most striking features of the baleen rack of MUSM 3292 is its apparent shortness, with baleen seemingly being absent along the anterior portion of the rostrum (Fig. 2B). There are several ways in which this unusual shortness might be explained. First, erosion could plausibly have obliterated parts of the rack prior to discovery, but we note that even along its most exposed and heavily weathered portion, traces of the plates remain clearly visible. Secondly, it is possible that the anterior portion of the rack simply was not preserved; however, the articulated state of the specimen suggests that the carcass was quickly covered and, barring the possible destruction of the rack by a scavenger prior to burial (itself unlikely, given that the rack is nearly *in situ*), similar fossilisation conditions presumably applied to the entire jaw. Thirdly, damage of the anterior portion of the rack could have occurred during preparation, but this is improbable because work on the right side of the jaw was largely confined to gentle brushing and consolidation.

**Figure 4:**
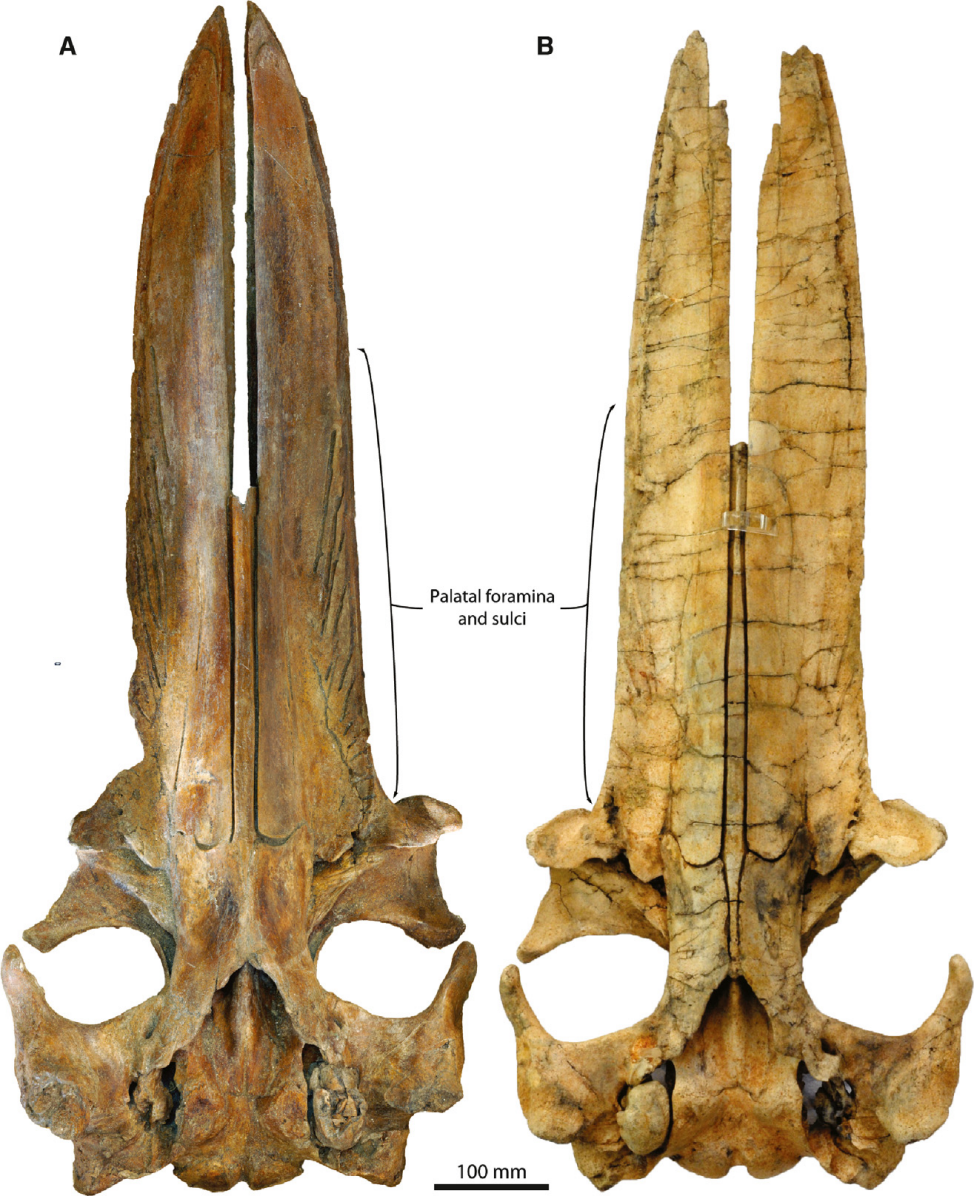
Distribution of palatal nutrient foramina and sulci in other specimens of *Piscobalaena nana*. (A) MNHN SAS1617; and (B) SMNK Pal4050 (holotype).

The final option – namely, that the rack is genuinely short – is supported by the fact that its size and position approximately match the area of the palate bearing nutrient foramina and sulci, which are well developed posteriorly, but absent anteriorly (Fig. 2B,D). A similar distribution of the sulci occurs in other specimens of *P. nana* (Fig. 4), but is otherwise a rare phenomenon restricted to the archaic eomysticetids, where it may similarly indicate the presence of a short rack (Boessenecker & Fordyce, 2015). A reduction of the anterior sulci also occurs in extant right whales, possibly because of their narrow maxilla; unlike in *Piscobalaena* and eomysticetids, however, faint anterior sulci often remain visible (e.g. *Balaena mysticetus*, IRSNB 1533; *Eubalaena glacialis*, USNM A23077), and large, anteriorly oriented nutrient sulci on the central portion of the palate suggest a well‐developed blood supply to the anterior portion of the rostrum. The complete absence of anterior sulci in *Piscobalaena* and eomysticetids may hence indeed suggest that the anterior rostrum lacked baleen (i.e. a large subrostral gap). In other cetotheriids, such as *Joumocetus* and *Cetotherium*, the palatal sulci extend closer to the tip of the maxilla, as in most crown mysticetes, suggesting that the condition in *Piscobalaena* may be derived.

### Implications for feeding

The detailed structure of baleen may be determined by a range of factors, including body size, phylogeny and function (Pivorunas, 1979; Young, 2012). Divergent patterns among extant species of comparable size (e.g. *Caperea* and *B. acutorostrata*), common descent (e.g. *B. borealis* vs. other balaenopterids), and similar feeding ecology (e.g. *Caperea* vs. right whales) suggest that baleen morphology often reflects a combination of all three (Young, 2012). In the features observable here – closely spaced plates and fine, dense tubules – the baleen of *Piscobalaena* most closely resembles that of *Caperea* (Young, 2012), although the two also markedly differ, for example in the size of the subrostral gap (larger in *Piscobalaena*) and, presumably, the length of the individual plates (markedly shorter in *Piscobalaena*). The similarities may partially be explained by small body size, although comparable features also occur in much larger species, such as right whales and *B. borealis*, and sometimes are absent in other relatively small whales (e.g. low bristle density in *B. acutorostrata*; Young, 2012). Alternatively, or in addition, the similarities between *Piscobalaena* and *Caperea* may reflect a common phylogenetic origin (Marx & Fordyce, 2016).

Among extant whales, the features characterising the baleen of *Piscobalaena* are typical of skim feeders (*Caperea*, right whales and *B. borealis*) targeting extremely small prey, such as copepods (Young, 2012). Skimming in these species is supported by an arched rostrum and elongate baleen to maximise the filtering surface (Brodie & Vikingsson, 2009; Werth & Potvin, 2016), as well as, in *B. borealis*, stiffening of the fine bristles via calcification (Szewciw et al. 2010). Similar adaptations for skimming are absent in platyrostral *Piscobalaena*, suggesting either that it captured small prey via a different approach, or that skim feeding on copepods was, at best, facultative. A different form of skim feeding could have been facilitated by the large subrostral gap and elongate rostrum, as proposed for eomysticetids (Boessenecker & Fordyce, 2015). In addition, restricted mandibular abduction and heighted control over longitudinal (alpha) rotation of the mandible, as in the closely related cetotheriid *Herpetocetus* (El Adli et al. 2014), could plausibly have contributed to fine‐tuning mandibular orientation during skimming. Nevertheless, the idea of *Piscobalaena* targeting diminutive prey is contradicted by the fossilised stomach contents of an unnamed, larger but phenetically similar Late Miocene cetotheriid from Peru, which seems to have fed on sardines (Collareta et al. 2015).

Contrary to previous suggestions (Bouetel & de Muizon, 2006), rorqual‐like lunge feeding on fish and/or krill can likely also be excluded, given that *Piscobalaena* differs from most balaenopterids in the morphology of its baleen (Young, 2012) and furthermore lacks relevant osteological adaptations, such as a thickened, pulley‐like postorbital ridge (Lambertsen et al. 1995; Marx & Kohno, 2016). Finally, the highly unusual mandibular morphology of cetotheriids, as well as reduced mandibular abduction and fine‐tuning of alpha rotation as mentioned above, has also been linked to suction feeding (El Adli et al. 2014; Gol'din et al. 2014). In *Piscobalaena*, this strategy could plausibly have been further facilitated by the large subrostral gap, although this feature is not obviously present in other cetotheriids (Gol'din et al. 2014). Moreover, the baleen of *Piscobalaena* markedly differs from that of the only extant mysticete known to use suction, the grey whale *Eschrichtius robustus* (Young, 2012; Young et al. 2015). Nevertheless, *Eschrichtius* is mainly a benthic feeder, and hence may show specialisations not seen in other, potentially more pelagic mysticetes. In addition, cetotheriids may have differed from *Eschrichtius* in using a form of continuous, rather than intermittent, suction feeding (Kimura, 2005; Gol'din et al. 2014). Whether and how such differences were reflected in the structure of the baleen currently remains unclear.

## Conclusion

Overall, the feeding strategy of *Piscobalaena* remains open to question, with the morphology of the baleen and skull seemingly at odds. The combination of fine, dense baleen with a mandibular morphology thought to reflect suction reveals a previously underappreciated degree of complexity in mysticete feeding evolution, and may point to ecological niches no longer occupied by living whales. Most importantly, the disparity of the feeding apparatus of *Piscobalaena* highlights the critical role of fossils and exceptional preservation in creating a comprehensive framework, within which the ecology and functional morphology of modern species can be understood.

## Supporting information


**Fig. S1.** Skull of *Piscobalaena nana* (MUSM 3292), in ventral view.

**Fig. S2.** Auditory region of *Piscobalaena nana* (MUSM 3292), in posterolateral view.
