# Supplementary figures and images for: How whales used to filter: exceptionally preserved baleen in a Miocene cetotheriid
Source: J Anat. 2017 May 24;231(2):212–20. doi: 10.1111/joa.12622 (PMC5522891; doi:10.1111/joa.12622)

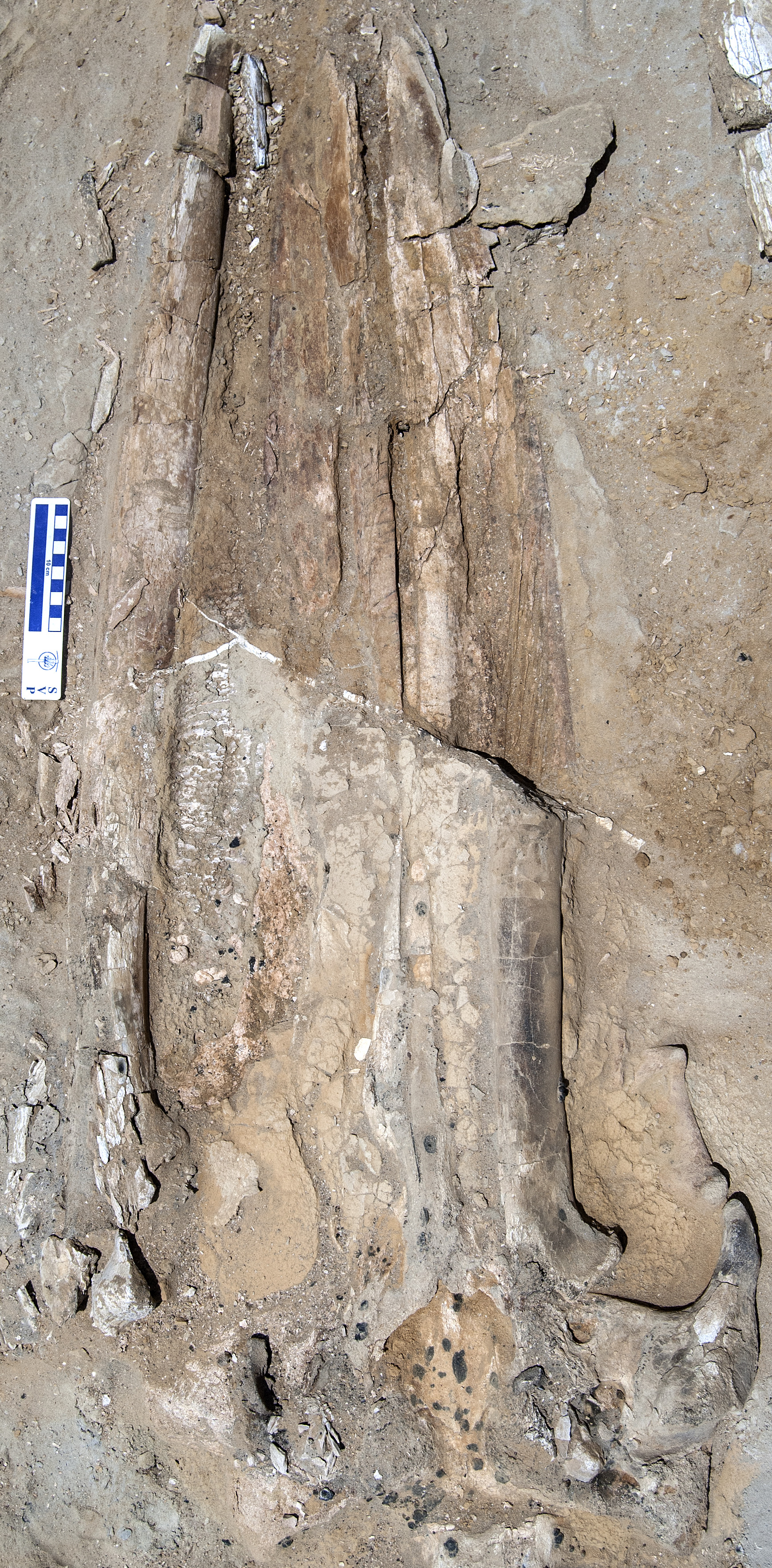

Supplement: Supplementary file 1 — Fig. S1. Skull of Piscobalaena nana (MUSM 3292), in ventral view. [file JOA-231-212-s001.jpg]

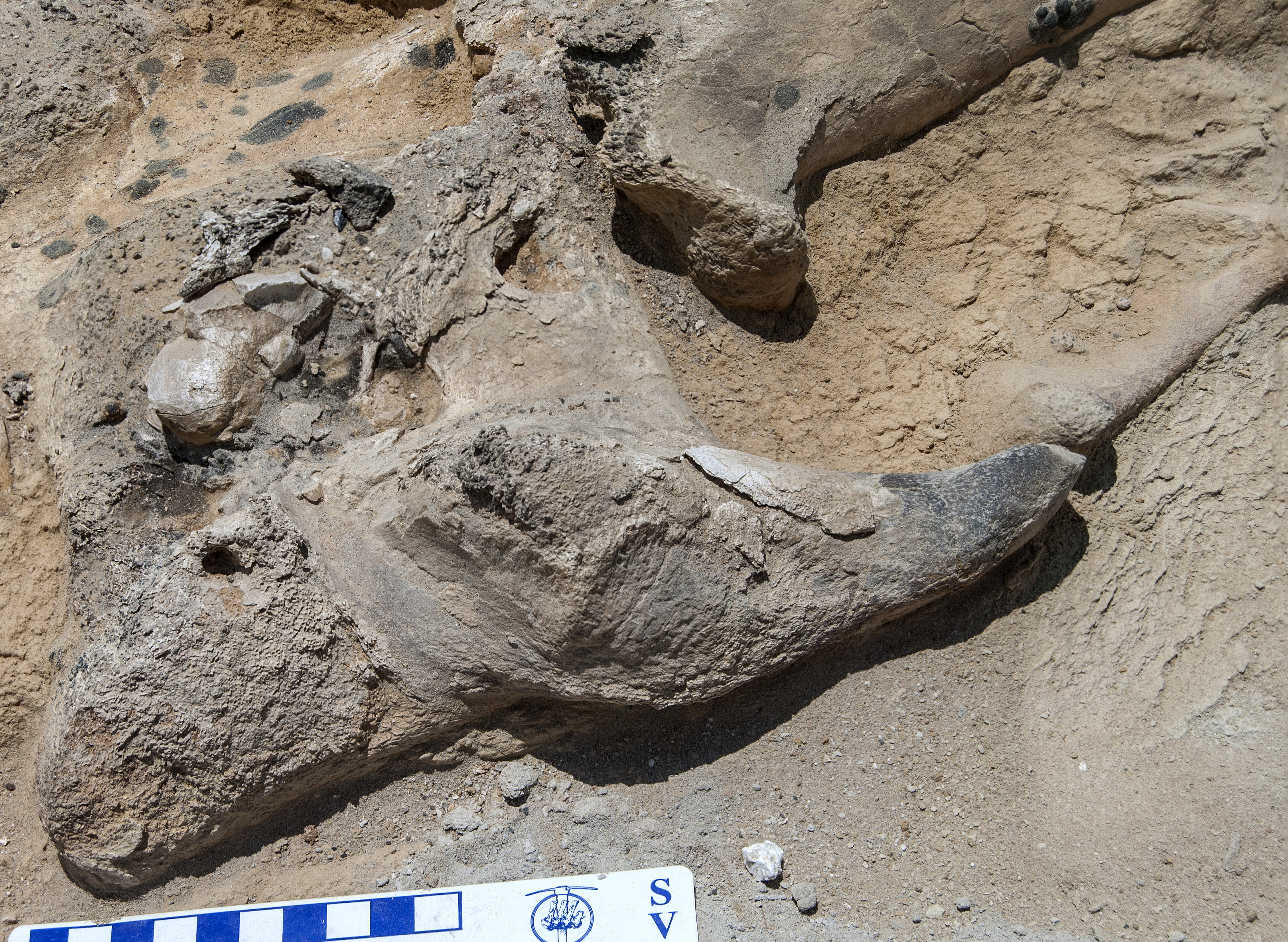

Supplement: Supplementary file 2 — Fig. S2. Auditory region of Piscobalaena nana (MUSM 3292), in posterolateral view. [file JOA-231-212-s002.jpg]
